# LiDAR-Based Long-Term Mapping in Snow-Covered Environments

**DOI:** 10.3390/s25216805

**Published:** 2025-11-06

**Authors:** Jaewon Lee, Woojin Chung, Jiwoong Kim

**Affiliations:** 1 Department of Mechanical Engineering, Korea University, Seoul 02841, Republic of Korea; 2 Purpose-Built Mobility Group, Korea Institute of Industrial Technology, Gwangju 61012, Republic of Korea

**Keywords:** LiDAR, SLAM, long-term mapping, snow-covered environments, autonomous driving

## Abstract

Autonomous driving systems encounter various uncertainties in real-world environments, many of which are difficult to represent in maps. Among them, accumulated snow poses a unique challenge since its shape and volume gradually change over time. If accumulated snow is included in a map, it leads to two main problems. First, during long-term driving, discrepancies between the actual and mapped environments, caused by melting snow, can significantly degrade localization performance. Second, the inclusion of large amounts of accumulated snow in the map can cause registration errors between sessions, thereby hindering accurate map updates. To address these issues, we propose a mapping strategy specifically designed for snow-covered environments. The proposed method first detects and removes accumulated snow using a deep learning-based approach. The resulting snow-free data are then used for map updating, and the ground information occluded by snow is subsequently restored. The effectiveness of the proposed method is validated with data collected in real-world snow-covered environments. Experimental results demonstrate that the proposed method achieves 78.6% IoU for snow detection and reduces map alignment errors by 12.5% (RMSE) and 15.6% (Chamfer Distance) on average, contributing to maintaining map quality and enabling long-term autonomous driving in snow-covered environments.

## 1. Introduction

As autonomous driving technology advances rapidly, extensive research has been carried out to ensure reliable operation under diverse environmental conditions [1,2,3]. LiDAR (Light Detection and Ranging) sensors, providing fast and accurate distance measurements, have been widely used as a key perception modality in autonomous driving research. Nevertheless, under adverse weather conditions such as heavy snowfall, LiDAR performance can be significantly degraded due to a reduced detection range and increased noise [4]. To address this issue, existing studies have focused on mitigating snowfall-induced noise in LiDAR measurements [5,6,7,8,9,10,11,12,13,14].

**Figure 1:**
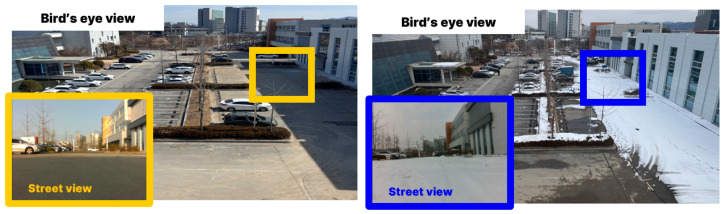
Comparison of the environment under snow-covered and snow-free conditions.

However, the influence of snow is not confined to measurement noise caused by snowfall. Snow accumulated on the ground can significantly affect autonomous driving performance. As shown in Figure 1, snow in real-world environments repeatedly accumulates and melts over time. A map generated under snowy conditions may not accurately represent the actual environment after the snow melts. This discrepancy can cause localization errors when the robot revisits the same area [15]. In addition, accumulated snow has a negative impact on map updating. In environments with frequent structural changes, such as urban areas or construction sites [16], continuous map updates are essential. This requires the robot to periodically revisit the same location and align newly acquired sensor data with the prebuilt map to reflect environmental changes. However, accumulated snow interferes with this process because it provides distorted information about the terrain. As a result, large-scale snow accumulation can be a significant source of registration errors during map updating.

This study addresses LiDAR-based long-term mapping techniques that can robustly operate in environments with frequent snowfall. Long-term mapping refers to periodically maintaining and updating maps by retaining static structures for reliable autonomous driving. To achieve this, we detect and remove temporally varying snow, thereby enabling more accurate detection of static environmental changes between maps. We collected LiDAR data under real-world snowy conditions and analyzed its physical characteristics. Based on these characteristics, we employ a deep learning-based snow detection algorithm to identify and remove snow from the map of the current session. To compensate for the sparsity of LiDAR scans, the proposed algorithm utilizes range-view images projected from the point cloud map instead of individual scans. The snow-removed map of the current session is then aligned with the map from the previous session for map updating. Through this process, registration errors caused by accumulated snow are reduced, thereby enabling more accurate detection of newly appeared or disappeared environmental features.

Subsequently, the ground information lost due to accumulated snow is restored. Without this step, the snow-removed regions may be misinterpreted as empty space and represented in the map as disappeared areas. Therefore, the proposed method preserves the actual terrain information through ground restoration.

The main contributions of this paper are as follows:

1. We develop a deep learning-based segmentation method that effectively detects snow by utilizing LiDAR reflectivity features and resolving input sparsity limitations.
2. We reduce registration errors between maps by utilizing snow-removed data, thereby maintaining accuracy in the map updating process.
3. We restore ground regions occluded by snow using information from the previous session, thereby preserving the overall consistency of the map.

## 2. Related Work

Most studies on LiDAR sensing in snowy environments have focused on the real-time filtering of snowfall-induced noise. This type of noise occurs when the sensor detects snow particles rather than actual terrain or objects, thereby degrading perception performance. To address this issue, various approaches have been proposed. Distance-based methods exploit the fact that snow particles tend to appear in isolation, whereas true object surfaces consist of densely arranged neighboring points [6,7,8]. Intensity-based methods rely on the weak intensity of snow, classifying low-intensity points as snowfall noise [9]. Several methods combine distance and intensity information to improve robustness [10,11]. In addition, learning-based methods have been proposed. 4DenoiseNet incorporates temporal and spatial coherence from consecutive LiDAR frames to distinguish noise [12]. LiSnowNet converts point clouds into range images and applies a tailored CNN architecture for snow removal [13]. SLiDE adopts a self-supervised learning strategy that enables training without labeled data, reducing annotation costs [14]. However, these studies primarily focus on falling snow rather than accumulated snow.

Meanwhile, several studies have investigated environments with accumulated snow. Some works focus on measuring or estimating snowfall amount and distribution. Wang et al. [17] combine satellite imagery and LiDAR data with a U-Net model to track spatiotemporal snow changes in complex mountainous terrain. MAPunet [18] integrates LiDAR maps, satellite imagery, terrain information, and meteorological data to predict per-pixel snow depth in mountain regions. In addition, Park et al. [19] apply LiDAR filtering to rapidly extract snow particles and classify snowfall intensity under diverse conditions, and the resulting information can be utilized to infer ground accumulation. Apart from measuring snow accumulation, some studies investigate its impact on robot operation. For example, Baril et al. [15] analyze repeated-route performance in severe winter forests as snow accumulates, while Boxan et al. [20] evaluate how seasonal variation and accumulated snow affect long-term localization accuracy. Despite these efforts, there is still limited research on mapping strategies tailored for autonomous driving in snow-accumulated environments.

To address environmental changes and ensure reliable autonomous driving, lifelong mapping strategies have been proposed, focusing on dynamic object removal, multi-session alignment, change detection, and map updating. Dynamic object removal is required to obtain maps that are robust to noise, since temporary objects such as vehicles or pedestrians degrade long-term usability. Removert [21], ERASOR [22], and Beautymap [23] removed such objects using geometric and positional information, while 4D-MOS [24] refined object separation by analyzing temporal motion patterns. Accurate alignment across sessions is essential for establishing a consistent reference frame for change detection over time. LT-mapper [25] combined anchor node-based SLAM, Scan Context place recognition, ICP, and visibility-based alignment to improve registration. ELite [26] introduced bidirectional alignment to mitigate short-term variations, and Yang et al. [27] proposed a two-stage method with feature-based initialization and fine registration for robustness across LiDAR sensors. To maintain map reliability, change detection becomes necessary since the map must continuously reflect changes. LT-mapper [25] distinguished new and disappeared structures while considering occlusions. ELite [26] evaluated changes based on their duration, and in [27], BEV projection and height analysis were applied for efficient large-scale detection. Map updating techniques have been developed to complement these approaches. LT-mapper [25] stored only changed regions for memory efficiency, ELite [26] prioritized updates by importance, and Yang et al. [27] introduced version control for temporal comparison and past map restoration. Nevertheless, these approaches remain limited in addressing temporary yet long-lasting environmental changes, such as accumulated snow.

## 3. LiDAR Characteristics on Snow-Covered Ground

To detect accumulated snow, we utilized the reflectance characteristics of LiDAR measurements collected over snow-covered ground. LiDAR intensity is inherently affected by both incidence angle and range [28]. While the effects of range can be compensated independently, those of the incidence angle are difficult to correct because they vary across surface materials. Nevertheless, Anttila et al. [29] proposed an empirical correction method for incidence angle effects on accumulated snow, where multiple scattering occurs. However, since our goal is to distinguish snow from other objects, surface properties are unknown a priori. Applying the snow-specific correction function from [29] to the entire point cloud would be inappropriate, as it could distort the intensity values of non-snow objects. Therefore, we leverage both intensity and reflectivity measurements from the Ouster OS1-64 LiDAR sensor, acquired from multiple viewpoints, for snow detection. Intensity represents the raw backscattered signal strength, whereas reflectivity is the manufacturer-calibrated value of intensity that compensates for range effects. Since the incidence-angle effects, which vary with surface properties, are difficult to accurately correct, we utilize multi-viewpoint intensity measurements acquired as the robot traverses the environment. This approach helps mitigate the uncorrected incidence-angle effects present in individual viewpoints.

In this section, asphalt pavement was used as the reference surface for non-snow conditions. Focusing on an area of approximately 60 m^2^, reflection characteristics were analyzed using accumulated point cloud maps generated with LIO-SAM [30], a LiDAR-inertial odometry and mapping framework. The Ouster OS1-64 LiDAR was used for data collection. Since single-scan data provide only sparse measurements, and snow typically accumulates over broad areas, it is difficult to capture reliable reflection patterns from a single frame. To address this, dense accumulated point clouds were employed, offering a more consistent basis for comparing snow-covered and snow-free surfaces.

**Figure 2:**
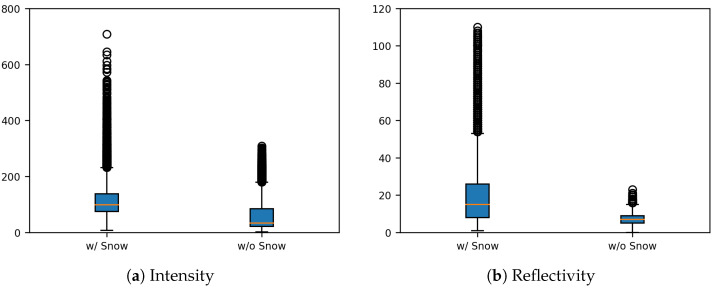
Boxplot comparison of LiDAR intensity and reflectivity between snow-covered and snow-free ground.

A comparison of intensity and reflectivity between snow-covered and snow-free surfaces in the point cloud map revealed distinct differences, with snow-covered regions showing higher mean values—approximately 53.6 for intensity and 11.9 for reflectivity. As illustrated in Figure 2, snow-covered areas generally occupied higher ranges across both indicators, with elevated medians and upper quartiles. Outliers were more prevalent in snow-covered regions, indicating irregular strong reflections, whereas snow-free ground exhibited narrower interquartile ranges and fewer outliers, reflecting more stable surface properties. These distinctions demonstrate that both intensity and reflectivity are effective physical cues for snow detection. Accordingly, this study proposes a detection method that leverages the dense representation of accumulated point clouds to capture such surface patterns.

## 4. Proposed Method

### 4.1. System Architecture

**Figure 3:**
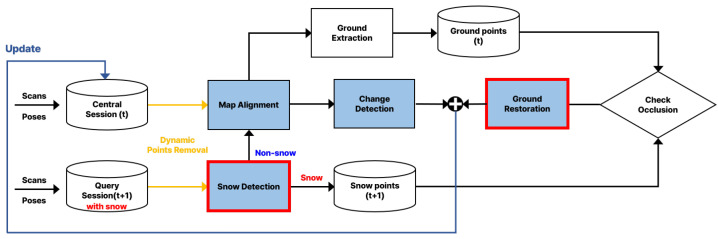
Overview of the proposed mapping system in snow-covered environments. The architecture consists of four main modules: Snow Removal, Map Alignment, Change Detection, and Ground Restoration.

This study proposes a system architecture for map updating in snow-covered environments. Figure 3 illustrates the overall flow of the proposed architecture. The architecture consists of four core modules: Snow Removal, Map Alignment, Change Detection, and Ground Restoration. The system first detects and removes snow from LiDAR point cloud data. It then performs inter-session registration to align multiple sessions. After alignment, environmental changes are identified by comparing the sessions. Finally, the system restores ground surfaces occluded by snow.

The proposed architecture adopts the modular framework of LT-mapper [25] and ELITE [26], which use sequential modules for dynamic object removal, alignment, and change detection. However, we make two key modifications for snow-covered environments. First, we develop and integrate a learning-based snow removal module that explicitly detects and removes accumulated snow from point cloud maps. Unlike dynamic objects, snow changes over time but appears static within a session and evades conventional dynamic filters, requiring explicit removal. Second, we introduce a ground restoration module that recovers terrain regions misclassified as disappeared due to occlusion by snow coverage, preserving actual ground surface information in the updated map.

The Central Session is defined as a session from previously constructed maps that serves as the reference for alignment during the update process. The Query Session, in contrast, is a newly collected session under snowy conditions. Both sessions are used as inputs to the proposed architecture, and their map data is constructed as point clouds through LIO-SAM [30]. Since both sessions were collected at the same location, they contain sufficient overlapping regions for alignment.

In each session, we first use the Removert module [21] included in the LT-mapper to remove highly dynamic (HD) points such as vehicles and people. These points move significantly within the individual session and thus appear in different positions across consecutive scans. HD points contribute less to the distinctiveness of a location compared to static structures. In addition, their motion often induces surrounding noise that degrades point cloud accuracy. Therefore, they are removed before the snow detection stage. After this preprocessing, in the Snow Removal stage, accumulated snow points within the Query Session are detected and removed. This is achieved using a deep learning-based range-view segmentation model that leverages intensity and reflectivity characteristics of snow.

In the Map Alignment stage, the snow-removed Query Session is aligned with the Central Session using various alignment algorithms. The typical approach first identifies overlapping regions between the two maps, then employs multi-session pose graph optimization (PGO) with scan-to-map ICP refinement to align the sessions within a unified coordinate frame. After registration is completed, the Change Detection stage of LT-mapper identifies regions of difference between the two sessions. These differences are referred to as Low Dynamic (LD) changes, which correspond to static environmental variations such as construction walls, trees, or parked cars. LD changes are further divided into

- Positive Dynamic (PD): Points newly observed in the Query Session that are added to the map.
- Negative Dynamic (ND): Points present in the Central Session but not observed in the Query Session, and thus removed from the map.

One challenge in the Change Detection stage is that ground points covered by snow may be misclassified as ND. This occurs because removing snow from the Query Session results in the loss of ground information, causing the ground to be mistakenly classified as absent when compared with the Central Session. To overcome this problem and ensure that true ground structures are preserved, the Ground Restoration stage is introduced. In this stage, ground surfaces are extracted from the Central Session, and are assessed to identify ground areas obscured by snow. The identified ground is then reintegrated into the updated map.

**Figure 4:**
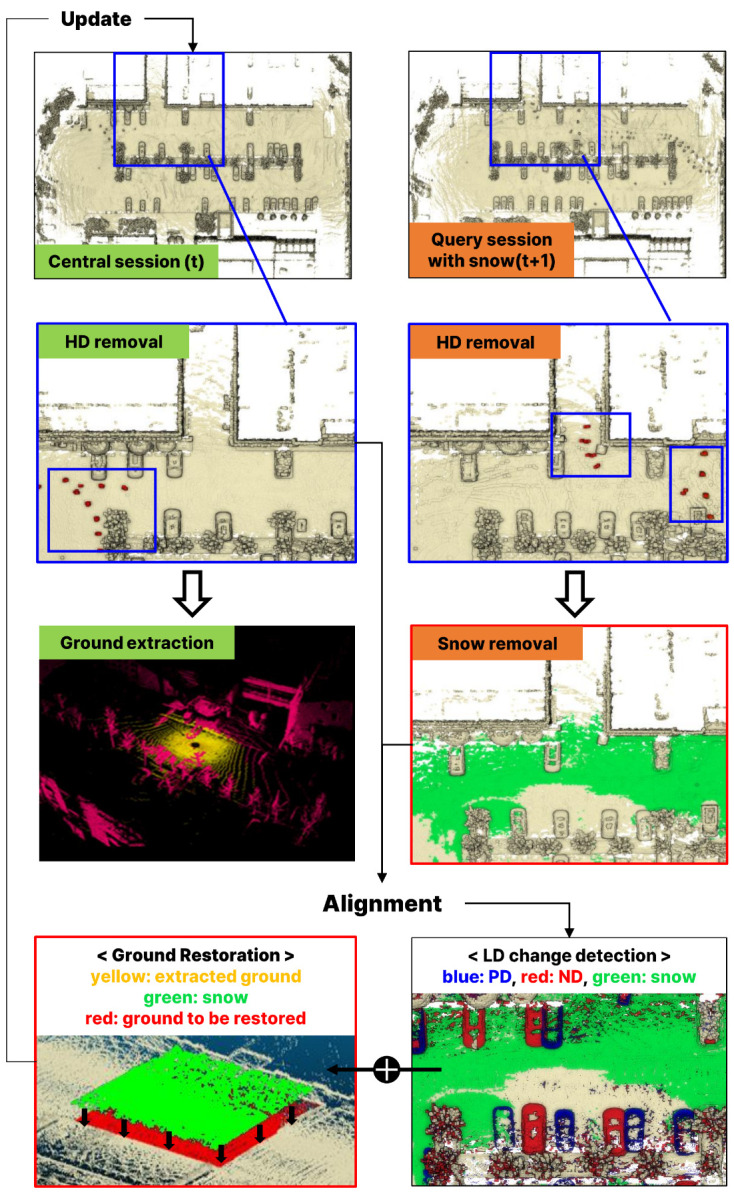
Visualizationof the proposed architecture [21,25,31].

Figure 4 visualizes the proposed system architecture.

### 4.2. Snow Detection Algorithm

**Figure 5:**
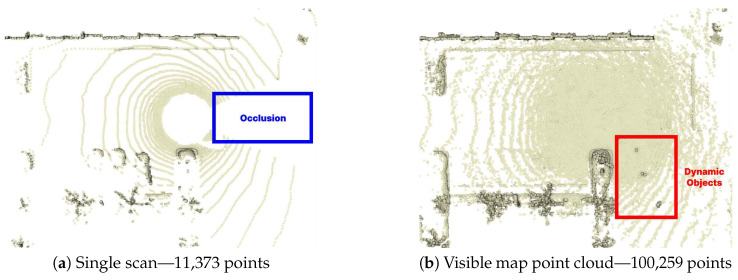
Comparison of single scan and visible map point cloud.

In snowy environments, accumulated snow appears temporarily but remains fixed in static positions, making it difficult to remove with conventional filters designed for dynamic objects. Moreover, as shown in Figure 5a, a single-scan point cloud suffers from a narrow field of view and low point density, which makes it challenging to adequately capture and distinguish widely distributed snow. To overcome these limitations, this study leverages the reflective characteristics of accumulated snow analyzed in Section 3 and applies a learning-based detection algorithm. The proposed method operates on accumulated point cloud maps rather than single scans.

In this study, we utilized the Visible Map Point Cloud defined in Removert to obtain high-density data required for training snow accumulation detection. This point cloud is generated from a map constructed by registering keyframes selectively stored during SLAM. The generation process is as follows. For each keyframe, the sensor is placed at its corresponding map position, and the visible point cloud is generated by re-observing the map from that perspective, applying the relative transformation to ensure accurate observation geometry. Additionally, the sensor’s field of view (FOV) is defined, and only the closest point in each viewing direction is selected.

More specifically, the integrated LiDAR map $P^{M}$ obtained from multiple scans is projected into a 2D range image $I_{k}^{M}\in R^{m\times n}$ through spherical projection, where *k* denotes the keyframe index. The range image resolution (m,n) determines the density of the projection, with higher resolutions enabling more map points to be captured.

The spherical projection process divides the sensor’s field of view into a grid of angular bins. For each pixel (i,j) in the range image, the corresponding viewing direction is defined by azimuth angle $\theta_{i}$ and elevation angle $\phi_{j}$. Each pixel represents an angular bin with extents Δθ and Δϕ in the horizontal and vertical directions. The set of map points $P_{ij}^{M}$ falling within this angular bin is defined as

(1) $$P_{ij}^{M}=\left\{p\in P^{M}\;|\;\theta_{i}-\frac{\Delta \theta }{2}\leq \theta \left(p\right)<\theta_{i}+\frac{\Delta \theta }{2},\phi_{j}-\frac{\Delta \phi }{2}\leq \phi \left(p\right)<\phi_{j}+\frac{\Delta \phi }{2}\right\}$$

where $\theta \left(p\right)=\arctan\left(y_{p}/x_{p}\right)$ and $\phi \left(p\right)=\arctan\left(z_{p}/\sqrt{x_{p}^{2}+y_{p}^{2}}\right)$ are the azimuth and elevation angles of point *p* relative to the keyframe’s coordinate system. Among all points in $P_{ij}^{M}$ that fall within the same angular bin, only the one with minimum range is retained and assigned to pixel (i,j). This mimics the physical behavior of LiDAR sensors, which detect only the nearest surface along each ray direction:

(2) $$I_{k,ij}^{M}=\min_{p\in P_{ij}^{M}}r\left(p\right),\;p_{k,ij}^{M}=\arg\min_{p\in P_{ij}^{M}}r\left(p\right)$$

where $r\left(p\right)=\sqrt{x_{p}^{2}+y_{p}^{2}+z_{p}^{2}}$ is the Euclidean distance from the keyframe to point *p*. The collection of all selected points forms the visible map point cloud:

(3) $$P_{k}^{M}=\left\{p_{k,ij}^{M}\right\}$$

This process effectively filters the dense global map $P^{M}$ to retain only the points visible from the keyframe’s perspective, accounting for occlusions. As illustrated in Figure 5b, the resulting visible map point cloud $P_{k}^{M}$ provides higher density than a single scan, and when combined with the dynamic object removal module, dynamic objects (highlighted by red boxes) can be eliminated to yield a cleaner point cloud. In addition, while single scans may suffer from occlusions caused by dynamic objects present at the time of scanning, such occlusions are effectively mitigated.

The point cloud projected from the map contains significantly more points than a single scan. As shown in Figure 5, it includes more than ten times the number of points. However, directly using such high-density point clouds for training substantially increases computational and memory costs as the number of points grows. To address this, we adopt a range-view projection, which converts point clouds into fixed-resolution 2D images. This approach preserves spatial structure while keeping the input size constant, thereby improving training efficiency and making it well-suited for GPU computation.

**Figure 6:**
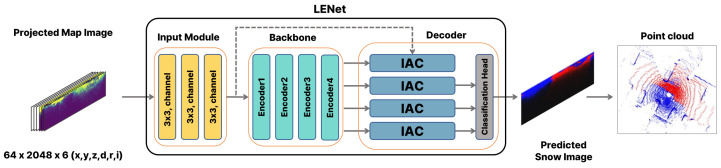
LENet-based network architecture for snow detection [32].

For learning-based accumulated snow detection, we build upon the range image segmentation model LENet [32]. Figure 6 illustrates the overall network framework adapted for snow detection, and Table 1 provides detailed specifications. LENet is a lightweight network that achieves a balance between efficiency and accuracy through an encoder based on Multi-Scale Convolutional Attention (MSCA) and a decoder based on Interpolation And Convolution (IAC). MSCA effectively captures multi-scale contextual information through convolutional operations with varying receptive field sizes, while IAC enhances representational power through bilinear interpolation for upsampling multi-resolution feature maps and integrating them via convolution without the need for a complex decoder.

**Figure 7:**
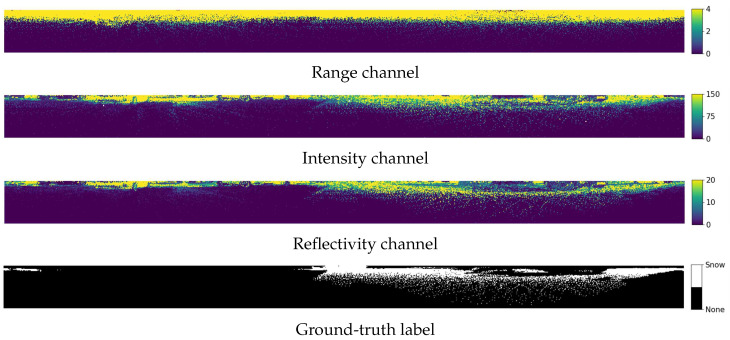
Input features and ground-truth labels for snow segmentation.

Figure 7 illustrates examples of the input images and snow region labels used for training. Based on keyframes containing snow, map-projected images with a resolution of 64×2048 were used as training inputs. Each image consists of six channels representing spatial coordinates (x,y,z), range (d), reflectivity (r), and intensity (i). Regions with high intensity and reflectivity show distributions similar to the annotated snow regions. This indicates that these features provide useful cues for snow detection.

The loss function in LENet is designed to jointly address class imbalance, boundary precision, and IoU optimization. Specifically, it combines Weighted Cross-Entropy Loss ($L_{wce}$), Lovász-Softmax Loss ($L_{ls}$), and Boundary Loss ($L_{bd}$). The main loss is defined as

(4) $$L_{main}=L_{wce}+1.5L_{ls}+L_{bd}.$$

In addition, auxiliary segmentation heads are attached to the last decoder stages, and their losses are computed together with the main loss to stabilize training and enhance overall performance. The overall loss function is given by

(5) $$L_{total}=L_{main}+\sum_{i=1}^{3}\lambda_{i}L\left(y_{i},{\hat{y}}_{i}\right),$$

where $y_{i}$ is the semantic output obtained from stage *i*, and ${\hat{y}}_{i}$ is the corresponding semantic label. In our implementation, we empirically set $\lambda_{1}=1$, $\lambda_{2}=1$, and $\lambda_{3}=0.5$.

The proposed method is applicable to environments where snow and ground surfaces exhibit distinguishable reflectivity characteristics, such as urban roads, parking areas, and paved driving surfaces. Additionally, our approach requires sufficient map point cloud density for reliable snow detection, as it leverages accumulated map points rather than single scans. Our implementation with Ouster OS1-64 uses the default keyframe generation thresholds (1.0 m translation or 0.2 radian rotation) set in LIO-SAM [30] for building the global map, but lower-resolution sensors may require more frequent keyframes to compensate for reduced point density.

### 4.3. Ground Restoration Algorithm

In multi-session map updating, the map is updated through change detection after inter-session alignment. To reduce snow-induced alignment errors, we removed the detected snow from the Query session, aligned it with the Central session, and performed change detection. As a result, the removed snow regions may be misinterpreted as missing when compared with the Central session, leading to ground being misclassified as ND (Negative Dynamic). To address this issue, we propose a Ground Restoration algorithm that recovers ground points misclassified as ND.

**Figure 8:**
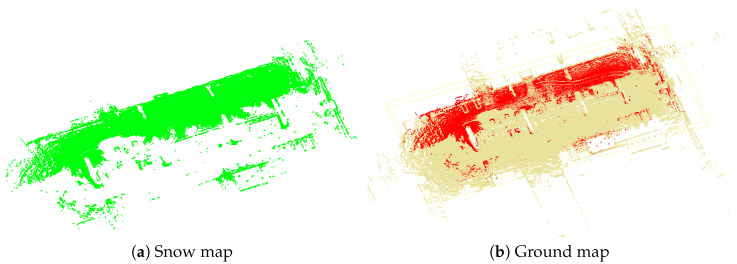
Comparison of snow map and ground map. Red points in the ground map indicate ND points misclassified by snow occlusion and selected as restoration targets.

First, we generate point clouds projected from the map for each keyframe of the Central session, and apply Patchwork [31] to extract ground surfaces at the keyframe level. Patchwork is a ground segmentation technique that partitions 3D LiDAR data into concentric ring-based regions, estimates ground planes in each region, and suppresses false positives using Ground Likelihood. Using the extracted keyframe ground surfaces from the Central session, we construct the ground map. Similarly, we construct the snow map using the snow regions obtained through snow detection in the Query session. Since the two sessions are aligned in the same coordinate system, we can directly compare the ground map and the snow map to identify the ground regions that need to be restored. Figure 8 illustrates the ground map and snow map constructed through this process.

The ground restoration procedure is described in Algorithm 1. This process is applied to each snow point in the snow map, where a voxel column is defined downward along the z-axis from the position of each snow point. Since LIO-SAM [30] aligns the z-axis with the gravity direction when establishing the initial coordinate system using IMU data, the z-axis in this coordinate system represents the gravity direction. The column length is set from each snow point to the lowest point in the ground map. If ground map points exist within this voxel column, the closest one to the snow point is selected as the restoration target. It is treated as ground temporarily occluded by snow and incorporated as restored ground during map updating. The red points shown in Figure 8b correspond to the restoration targets.
**Algorithm 1** Ground Restoration Algorithm**Require:** Snow point cloud $P_{\mathrm{snow}}$, ground point cloud $P_{\mathrm{ground}}$, vertical voxel size δ**Ensure:** Restored ground points $P_{\mathrm{restore}}$  1: $P_{\mathrm{restore}}\leftarrow \emptyset$  2: $z_{\min}\leftarrow \min\left\{z|\left(x,y,z\right)\in P_{\mathrm{ground}}\right\}$  3: **for all** $p_{s}=\left(x_{s},y_{s},z_{s}\right)\in P_{\mathrm{snow}}$ **do**  4:     $V_{p_{s}}\leftarrow \{\left(x,y,z\right)\in P_{\mathrm{ground}}||x-x_{s}|<\delta ,|y-y_{s}\left|<\delta ,z_{\min}\leq z<z_{s}\right\}$  5:     **if** $V_{p_{s}}\neq \emptyset$ **then**  6:         $p_{g}^{*}\leftarrow \arg\min_{\left(x,y,z\right)\in V_{p_{s}}}\left|z_{s}-z\right|$  7:         $P_{\mathrm{restore}}\leftarrow P_{\mathrm{restore}}\cup \left\{p_{g}^{*}\right\}$  8:     **end if**  9: **end for**10: **return** $P_{\mathrm{restore}}$

## 5. Experiments

### 5.1. Experimental Setup

**Figure 9:**
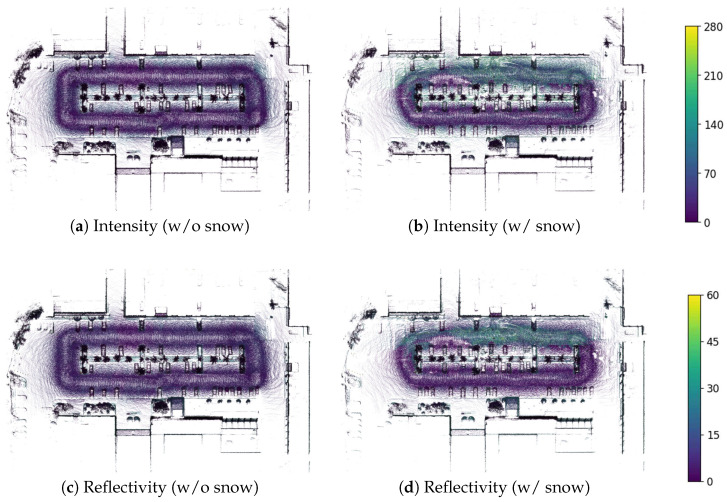
Visualization of intensity and reflectivity for environments without snow (**a**,**c**) and with snow (**b**,**d**).

To demonstrate the effectiveness of the proposed method, we conducted experiments using a Clearpath Jackal UGV equipped with an Ouster OS1-64 LiDAR sensor. The OS1-64 provides 64 vertical channels with a horizontal resolution of 1024 points per rotation and a maximum detection range of 120 m. Data collection was conducted in an outdoor parking lot in February 2025, covering both snow-covered and snow-free conditions. A total of four sessions and 824 keyframes were collected. Two snow-covered sessions were recorded under different snow depth conditions (2–3 cm and 5–8 cm) to capture variations in snow accumulation for training the snow detection network. In both cases, the snow exhibited varying conditions with some areas deformed by vehicle traffic and others remaining undisturbed. These snow-covered sessions were conducted under cloudy conditions at sub-zero temperatures (average −1.4 °C), ensuring that the accumulated snow remained stable without significant melting during data collection. Figure 9 illustrates the visualized intensity and reflectivity for both the snow-free and snow-covered sessions.

The dataset consisted of 600 samples, which were split into training (70%), validation (20%), and test (10%) sets while keeping the snow pixel ratio consistent across the splits. During the training process, data augmentation was applied using random rotation, random point dropout, and flipping of the 3D point cloud.

Model training was conducted using PyTorch 2.4.1 on an NVIDIA GeForce RTX 4070 Laptop GPU (8 GB, CUDA 12.4), with detailed hyperparameters provided in Table 2. After training, the model was converted into TorchScript format for compatibility with the C++-based map updating module and integrated with LibTorch to perform snow detection and map updating.

### 5.2. Snow Detection Results

Snow detection performance was evaluated using the Intersection over Union (IoU) metric, which is widely used for quantitatively comparing the performance of object detection and segmentation. IoU is defined as follows:

(6) $$\mathrm{IoU}=\frac{\mathrm{TP}}{\mathrm{TP}+\mathrm{FP}+\mathrm{FN}}$$

where TP, FP, and FN denote the number of true positive, false positive, and false negative predictions, respectively.

**Figure 10:**
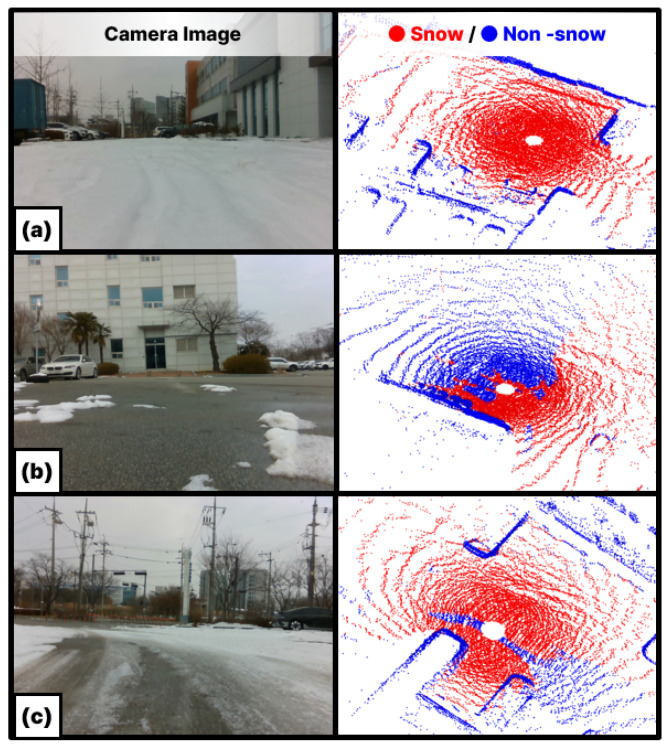
Snow detection results. (**a**) Fully snow-covered area (**b**) Clear snow-ground boundary (**c**) Ambiguous boundary.

**Figure 11:**
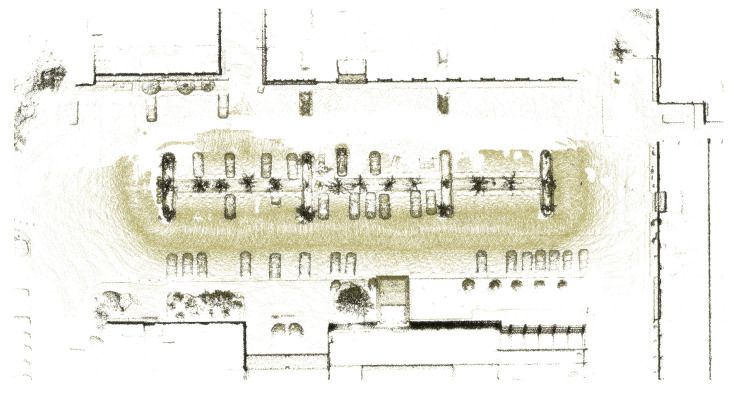
Map after snow removal.

Figure 10 shows the snow segmentation results obtained using intensity and reflectivity as input features. The model achieved an IoU of 78.6%. Stable performance was observed when the boundary between snow and ground was clearly distinguishable. However, in cases where the boundary was ambiguous, as illustrated in Figure 10c, misclassification occurred and the IoU for the snow class was reduced to 62%. Figure 11 presents the map after snow removal based on the detection results. This map was subsequently used as input for the map alignment process.

### 5.3. Map Alignment Results

This subsection analyzes the impact of snow removal on map alignment error. Following ELite [26], we evaluated multi-session map alignment performance using two key metrics: RMSE and Chamfer Distance (CD). To compute these metrics, we first identify point correspondences between two point clouds through nearest neighbor search and extract the inlier set using a distance threshold of 0.5 m. The RMSE metric quantifies the alignment error as the root mean squared distance between matched inlier pairs. The CD metric assesses alignment quality by computing the bidirectional sum of average inlier distance. The Chamfer Distance (CD) is formally defined as follows:

(7) $$\mathrm{CD}\left(X^{\prime },Y^{\prime }\right)=\frac{1}{\left|X\right|}\sum_{x\in X}\min_{y\in Y^{\prime }}{\left|x-y\right|}^{2}+\frac{1}{\left|Y\right|}\sum_{y\in Y}\min_{x\in X^{\prime }}{\left|y-x\right|}^{2}$$

Here, $X^{\prime }$ and $Y^{\prime }$ denote the complete sets of the two point clouds being compared, while *X* and *Y* represent the subsets of points whose nearest-neighbor distances in the opposite point cloud are within a given threshold. In this study, the threshold was set to 0.5 m.

To comprehensively validate the effectiveness of the proposed method, we evaluated it using a diverse set of registration algorithms: LT-SLAM [25], SLAM2REF [34], ELITE [26], VGICP [35], and NDT [36]. For VGICP, we configured a maximum correspondence distance of 1.5 m and 20 nearest neighbors for covariance estimation. NDT employed a voxel resolution of 1.0 m and a neighbor search radius of 2.0 m. Both methods ran for up to 64 iterations with convergence thresholds of $2\times 10^{-3}$ rad and $5\times 10^{-4}$ m for rotation and translation, respectively. The remaining methods (LT-SLAM, SLAM2REF, and ELITE) were configured with their default parameters from the original implementations.

**Figure 12:**
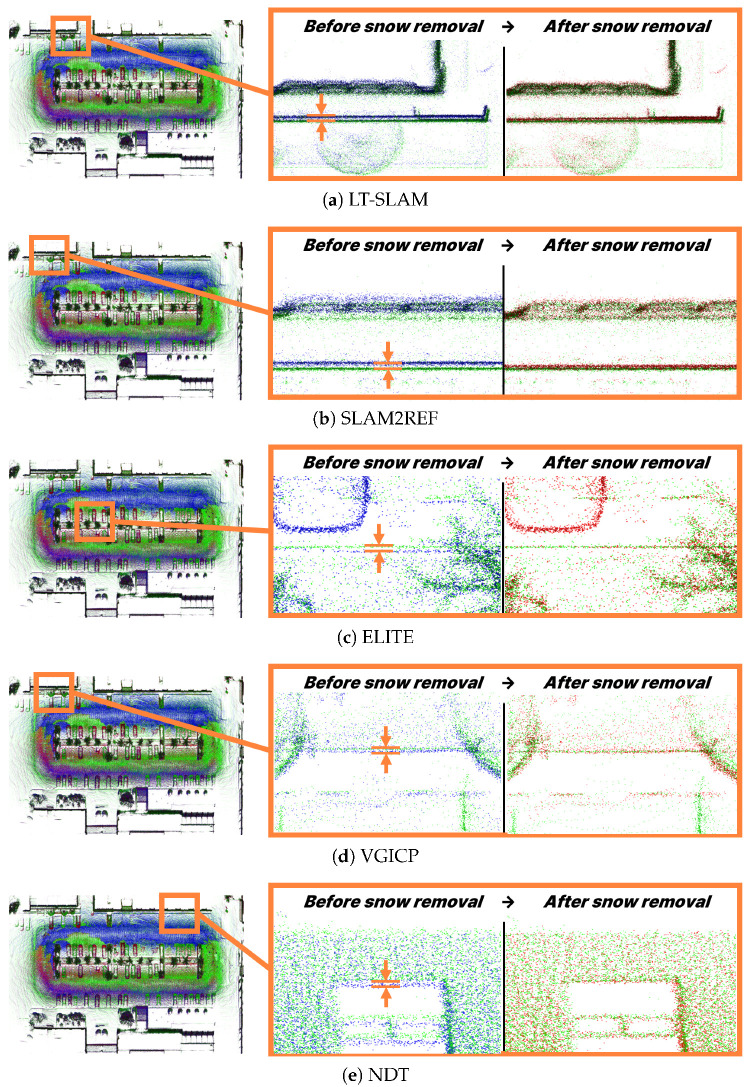
Map alignment results. Green: Central session, Blue: Query session before snow removal, Red: Query session after snow removal.

Table 3 summarizes map alignment errors for each method, and Figure 12 presents the visualized alignment results before and after snow removal. The results show a consistent reduction in alignment errors after snow removal across all methods.

### 5.4. Ground Restoration Results

**Figure 13:**
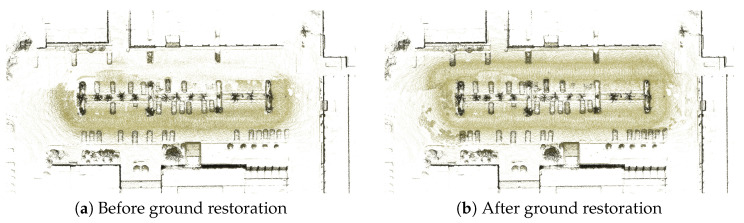
Ground restoration results.

In this section, we validate the effectiveness of the proposed ground restoration algorithm by comparing maps before and after snow removal. Figure 13a shows the map updated after snow removal. The lower ground beneath the snow was not reflected, resulting in a large vacant region. This deficiency arises because the change detection process misinterprets the area as occluded ground, which ultimately reduces the usability of the map.

As shown in Figure 13b, applying the proposed algorithm restores most of the occluded ground in the vacant region. Quantitatively, 95.30% of the ground regions that required restoration due to snow occlusion were successfully recovered using the Central session’s ground map. This result demonstrates that the proposed algorithm plays a crucial role in reliably preserving ground information.

## 6. Conclusions

In this paper, we propose a LiDAR-based mapping system for long-term navigation in snow-covered environments. To address the sparsity of single LiDAR scans, the proposed system employs range-view projection. Furthermore, snow points are detected and removed using a range-view semantic segmentation network that incorporates intensity and reflectivity. Experimental results demonstrated reliable snow detection performance, and map registration quality was improved as evidenced by reductions in RMSE and Chamfer Distance across multiple alignment methods. In addition, the proposed ground restoration algorithm compensated for the loss of ground information caused by snow removal and preserved the structural consistency of the map. Therefore, the proposed system establishes a reliable foundation for long-term autonomous driving in real-world winter environments. In future work, we will focus on extending the proposed method to enhance robustness under more diverse weather and terrain conditions.

## Figures and Tables

**Table 1:** Specifications of the network.

Module	Stage	Channels	Kernel/Stride
Input Module	Layers 1–3	6→8→16→32	3 × 3, s = 1
Encoder	Stage 1 (Resolution: 1×)	32	3 × 3, s = 1
(MSCA)	Stages 2–4 (1/2×, 1/4×, 1/8×)	32	3 × 3, s = 2
Decoder	Stage 1 (Concat)	32 + 32→16	3 × 3, s = 1
(IAC)	Stages 2–4 (Upsample + Concat)	16 + 32→16	3 × 3, s = 1
Head	Fusion (Concat 3 stages) → Output	48→16→2	1 × 1

**Table 2:** Training hyperparameters.

Parameter	Value
Optimizer	AdamW [33]
Initial Learning Rate	2 × $10^{-3}$
LR Scheduler	Cosine Annealing
Batch Size	4
Epochs	50
Activation	LeakyReLU

**Table 3:** Map alignment errors before and after snow removal (lower is better). Values are shown as before -> after with relative reduction in parentheses.

Method	RMSE (m)	Chamfer Distance (m)
LT-SLAM	0.146 -> 0.137 (−6.2%)	0.241 -> 0.226 (−6.2%)
SLAM2REF	0.138 -> 0.118 (−14.5%)	0.228 -> 0.186 (−18.4%)
ELITE	0.128 -> 0.108 (−15.6%)	0.206 -> 0.162 (−21.4%)
VGICP	0.126 -> 0.109 (−13.5%)	0.199 -> 0.169 (−15.1%)
NDT	0.126 -> 0.110 (−12.7%)	0.204 -> 0.170 (−16.7%)

## Data Availability

The datasets presented in this article are not readily available because they are part of an ongoing study and are subject to institutional restrictions. However, representative range-view LiDAR samples, including both snow-covered and snow-free scenes, along with their corresponding annotations, have been made publicly available at https://doi.org/10.5281/zenodo.17491204 (accessed on 1 November 2025). Requests to access the full dataset should be directed to the corresponding author.
